# The descriptive analysis of depressive symptoms and White Blood Cell (WBC) count between the sexual minorities and heterosexual identifying individuals in a nationally representative sample: 2005–2014

**DOI:** 10.1186/s12889-022-14847-6

**Published:** 2023-02-09

**Authors:** Junjie Lu, Jiarui Yang, Jingyang Liang, David Mischoulon, Maren Nyer

**Affiliations:** 1 Department of Social and Behavior Sciences, Harvard T.H. Chan School of Public Health, Boston, USA; 2 Department of Radiology, Massachusetts General Hospital, Harvard Medical School, Boston, USA; 3 Department of Tropical Medicine, School of Public Health and Tropical Medicine, Tulane University, New Orleans, USA; 4 Depression Clinical & Research Program, Massachusetts General Hospital, Harvard Medical School, Boston, USA

**Keywords:** Depression, Sexual minority, White Blood Cell Count

## Abstract

**Background:**

Sexual minorities are at a higher risk of suffering from depressive symptoms compared with heterosexual individuals. Only a few studies have examined the conditions of having depressive symptoms within different sexual minority groups, especially people with sexual orientation uncertainty in a nationally representative sample. Furthermore, few studies have explored whether the mean white blood count (WBC) is different between people with and without depressive symptoms among different sexual minority groups in a nationally representative sample.

**Methods:**

We analyzed the National Health and Nutrition Examination Survey (NHANES) data from 2005 to 2014 with a sample of 14,090 subjects. We compared the prevalence of depressive symptoms in subpopulations stratified by sex, sexual minority status, and race. We also examined the difference in mean WBC count between depressed and non-depressed people among heterosexual individuals and different sexual minority groups. Additionally, two multivariable logistic regression models were used to explore the association between sexual minority status and depressive symptoms, treating sexual minority status as both a binary and categorical variable.

**Results:**

Female sex (OR: 1.96, 95% CI: 1.72—2.22) and sexual minority status (OR: 1.79, 95% CI: 1.47—2.17) were both independently associated with depressive symptoms. Within the sexual minority population, subjects who were unsure about their sexual identities had the highest odds of having depressive symptoms (OR: 2.56, 95% CI: 1.40—4.68). In the subgroup analysis considering intersectionality, black sexual minority females had the highest rate of depressive symptoms (19.4%, 95% CI: 7.72—40.98). Finally, the mean WBC count differed significantly between people with and without depressive symptoms among male heterosexual individuals, female heterosexual individuals, and female sexual minorities, but not among male sexual minorities.

**Conclusions:**

Based on sex, race, and sexual minority status, black females of sexual minority status had the highest rate of depressive symptoms. Within sexual minority groups, participants who were unsure about their sexual identities had the highest odds of having depressive symptoms. Finally, the mean WBC count was significantly higher among people with depressive symptoms than those without depressive symptoms only among male heterosexuals, female heterosexuals, and female sexual minorities, but not among male sexual minorities. Future research should investigate the social and biological mechanisms of the differences.

**Supplementary Information:**

The online version contains supplementary material available at 10.1186/s12889-022-14847-6.

## Background

Sexual minorities are defined as those who self-identify as gay/lesbian, bisexual, not sure about their sexual identities, or other sexual orientations/identities [1]. Sexual minorities are prone to more mental and physical health issues compared to their heterosexual counterparts, including higher stress levels [2], higher suicide rates [3, 4], and elevated alcohol and drug use [5].

Depression is a major public health issue in the United States. In 2019, 18.5% of adults reported symptoms of depression that were mild, moderate, or severe in the past 2 weeks [6]. Prior research has indicated elevated levels of depression among sexual minorities [7–9]. Several risk factors for sexual minorities’ depressive symptoms include childhood adverse experiences [10], peer violence against people of their sexual orientations [11], and hate crimes [12]. The minority stress model is widely used to explain the adverse conditions introduced to sexual minorities [13, 14]. Sexual minority status imposes additional stress level on stigmatized individuals, including vicarious stress responses that can result from insults to the individual’s collective identity. Although several studies included people whose sexual orientation is uncertain in the UK, few studies have investigated this population in the US, especially on a national population scale [15, 16]. The subgroups within sexual minorities have also not been separately studied regarding if they differ in frequency of depression and severity of depressive symptoms.

Some recent studies have also demonstrated an association between depressive symptoms and inflammatory biomarkers [17, 18]. The change in inflammatory reaction level might be caused by the interaction among the innate and adaptive immune systems, neurotransmitters, and neurocircuits [17, 19]. Researchers have proposed that systemic inflammation is a primary biobehavioral pathway linking sexual and gender stigma to physical health outcomes [20]. Meanwhile, based on the Minority Stress Theory, both the source and the coping strategies of the stressors among sexual minority individuals might lead to the disadvantaged health conditions on the target population. Specifically, they are exposed to excess social stress due to the stigmatized status assigned to their identities by society, for example, devaluation, rejection, disrespect, and even hate crimes. Meanwhile, they may also experience limited access to stress-ameliorating resources (coping, social support) compared to their heterosexual peers because of social exclusion and marginalization [21]. Influenced by the social level factors, individuals are likely to experience depressive symptoms, which triggers the biological interaction between physiological and psychological process, leading to a higher inflammation biomarker level [22, 23]. However, the association was examined only for C reactive protein (CRP) and did not include white blood cell count (WBC), which is a primary and more widely measured inflammatory biomarker [24, 25].

In this study, we aim to 1) use descriptive analysis to examine the prevalence of having depressive symptoms within different sexual minority subgroups, including those who are not sure about their sexual identities; and 2) compare the mean WBC count between people with and without depressive symptoms among heterosexual and sexual minority identifying individuals.

## Methods

### Study sample and population

The National Health and Nutrition Examination Survey (NHANES) is a cross-sectional program designed to assess health and nutritional status of adults and children in the United States [26]. The sample was selected to represent the U.S. population of all ages. The NHANES interview includes demographic, socioeconomic, dietary, and health-related questions, while the examination component consists of medical, dental, and physiological measurements and laboratory tests [27].

In this study, we combined the NHANES data from 2005 to 2014, resulting in a sample of 23,065 individuals. We then excluded participants aged less than 20 years or older than 59 years, since they were not asked to respond to sexual behavior and smoking behavior questionnaires. After dropping observations with missing values on any of the variables used for analysis, we performed our complete analysis on 14,090 eligible subjects.

This study was purely observational and only used publicly available secondary data. Furthermore, all observations were de-identified. The NHANES Institutional Review Board (IRB) approved the NHANES program and the NCHS Research Ethics Review Board (ERB) after 2003. No other IRB was required for this study.

### Measurements

#### Sexual minority status

Participants were asked about their sexual orientation, and those who self-identified as gay/lesbian, bisexual, not sure about sexual orientation, and other sexual orientations were considered as sexual minorities in the study. People who self-identified as heterosexuals were not considered as sexual minorities.

#### Depressive symptoms

The Patient Health Questionnaire (PHQ-9) was used to measure depressive symptoms. The PHQ-9 includes nine questions that measure depressive symptoms; each question was scored from 0 (not at all) to 3 (nearly every day), resulting in a total score range from 0 to 27. Participants who scored more than 10 were considered to have depressive symptoms [28].

#### White blood cell (WBC) count

The Beckman Coulter method was used to measure the WBC count. The Beckman Coulter method of sizing and counting particles detects measurable changes in electrical resistance produced by nonconductive particles suspended in an electrolyte solution. Beckman Coulter counted and sized individual particles at a rate of several thousand per second. This method was independent of particle shape, color, and density [29]. In this study, we used the counter with the unit of 1000 cells/ul.

#### Covariates

Participants’ race was categorized as non-Hispanic White, Black, Hispanic, and other races. Other races included Mexican American, Asian, other races, and multi-racial. Education levels were categorized as Bachelor of Arts (B.A.) and above, Associate of Arts (A.A.) and some college, high school graduate, and never completed high school. Income categories were generated by the poverty-income ratio (PIR), which was a ratio of family income to poverty. Specifically, we divided the income level into 4 categories, including poverty (PIR < 1), low income (1.0 <  = PIR < 2.0), middle income (2.0 <  = PIR < 4.0), and high income (PIR >  = 4.0). Smoking status was categorized as smokers, former smokers, and never-smokers. Body mass index (BMI) was used to gauge the weight status of participants. BMI was categorized by underweight (< 18.5 kg/m^2^), normal (18.5–24.9 kg/m^2^), overweight (25.0–29.9 kg/m^2^), and obese (≥ 30.0 kg/m^2^). We also included the survey year into the data analysis to capture the possible time-based trends.

### Statistical analysis

Using descriptive statistics, the proportions of each variable were weighted by the study design, including the sample weight, the cluster, and the strata. By weighing the sample, we took the oversampling and complexity of the study design into consideration and made the results nationally representative. To compare different variables, Rao-Scott Chi-square tests were used for categorical variables, and t-tests were used for continuous variables.

We included several covariates in the multivariable logistic regression models to control for confounding effects, and we provided the directed acyclic graph for the study in the Supplementary table. Two models were fitted to examine such associations: one treated sexual minority status as a binary variable (sexual minority or not), and the other further divided sexual minority members into subgroups as a categorical variable. The coefficients of the variables from these fitted models were defined to be statistically significant if the two-sided *P*-value was less than 0.05. The data analysis was conducted using Stata/SE Version 16 (College Station, TX).

## Results

### Demographic characteristics

We examined a sample of 14,090 people in our analysis. After weighing the sample, the sample could represent the demographic characters of 131,958,349 people in the United States. Among the overall sample, 94.1% (95% CI: 93.5–94.8) were heterosexual, and 5.9% (95% CI: 5.2–6.5) belonged to sexual minorities. NHANES asked the self-identified sex of participants, and the answers include male and female. Stratified by biological sex assigned at birth, we found a significant difference in the proportion of the sexual minority sample: 4.8% (3.9–5.8) of males identified themselves as sexual minorities, whereas 6.9% (95% CI: 6.2–7.7) of females identified themselves as sexual minorities. We also found a difference in the proportion of people who had depressive symptoms stratified by biological sex assigned at birth, with 8.0% (95% CI: 7.4–8.7), 5.6% (95% CI: 4.9–6.4), and 10.4% (95% CI: 9.5–11.4) in the overall population, males, and females, respectively. These results are summarized in Table 1.

**Table 1:** The weighted demographic characters of the study sample stratified by biological sex, NHANES 2005 to 2014

Variable	Overall *n* = 14,090	Males *n* = 6,880	Females *n* = 7,201	*p*-value
	Proportion (95% Confidence interval)	Proportion (95% Confidence interval)	Proportion (95% Confidence interval)	
**Depressive symptoms **^**a**^				< 0.001 ^f^
No	92.0% (91.3–92.6)	94.4% (93.6–95.1)	89.6% (88.6–90.5)	
Yes	8.0% (7.4–8.7)	5.6% (4.9–6.4)	10.4% (9.5–11.4)	
**Sexual Minority status **^**b**^				< 0.001 ^f^
Heterosexual	94.1% (93.5–94.8)	95.2% (94.2–96.1)	93.1% (92.3–93.8)	
Sexual minorities	5.9% (5.2–6.5)	4.8% (3.9–5.8)	6.9% (6.2–7.7)	
**Sexual Orientation**				< 0.001 ^f^
Heterosexual	94.2% (93.5–94.8)	95.2% (94.2–96.1)	93.1% (92.3–93.8)	
Gay/lesbian	1.9% (1.5–2.4)	2.5% (1.8–3.5)	1.2% (0.9–1.7)	
Bisexual	2.8% (2.4–3.1)	1.4% (1.1–1.8)	4.1% (3.6–4.7)	
Not sure	0.8% (0.6–1.0)	0.5% (0.3–0.7)	1.1% (0.9–1.4)	
Others	0.4% (0.3–5.8)	0.3% (0.2–0.6)	0.5% (0.03–0.07)	
**Age **^**c**^				0.3 ^f^
< 40	48.9% (47.4–50.4)	49.5% (47.7–51.2)	48.4% (46.6–50.2)	
> = 40	51.1% (50.0–52.6)	50.5% (48.8–52.3)	51.3% (49.8–53.4)	
**Race**				< 0.001 ^f^
White	68.0% (64.9–71.0)	68.1% (65.1–71.0)	67.9% (64.5–71.1)	
Black	11.2% (9.6–12.8)	10.2% (8.9–11.7)	12.1% (10.3–14.1)	
Hispanic	5.3% (4.3–6.5)	5.2% (4.2–6.5)	5.3% (4.3–6.5)	
Other ^d^	15.6% (13.9–17.5)	16.5% (14.6–18.5)	14.7% (13.0–16.6)	
**Education**				< 0.001 ^f^
B.A. and above	30.3% (28.2–32.6)	28.7% (26.5–31.0)	32.0% (30.0–34.5)	
A.A. and some college	33.2% (32.0–34.3)	31.3% (30.0–32.7)	35.0% (33.5–36.5)	
High school	22.0% (20.6–23.4)	24.1% (22.5–25.8)	19.8% (18.4–21.4)	
Did not complete high school	14.5% (13.2–15.9)	15.9% (14.4–17.5)	13.2% (11.9–14.7)	
**Income **^**e**^				< 0.01 ^f^
High	37.9% (35.6–40.2)	38.8% (36.5–41.1)	37.0% (34.6–39.4)	
Middle	28.4% (27.0–30.0)	28.4% (26.8–30.1)	28.5% (26.9–30.1)	
Low	18.6% (17.5–19.8)	18.5% (17.3–19.9)	18.6% (17.5–19.9)	
Poverty	15.1% (13.7–16.5)	14.2% (12.8–15.8)	15.9% (14.4–17.5)	
**Smoking status**				< 0.001 ^f^
Non-smokers	55.8% (54.2–57.5)	51.5% (49.6–53.5)	60.1% (58.1–62.0)	
Former smokers	19.7% (18.6–20.7)	21.7% (20.4–23.0)	17.7% (16.2–19.2)	
Current smokers	24.5% (23.1–26.0)	26.8% (25.1–28.5)	22.2% (20.7–23.9)	
**BMI**				< 0.001 ^f^
Underweight	1.6% (1.3–1.9)	1.0% (0.7–1.3)	22.5% (18.6–27.1)	
Normal	30.1% (28.8–31.5)	26.2% (24.7–27.7)	34.1% (32.4–35.8)	
Overweight	32.3% (31.2–33.4)	38.3% (37.0–39.6)	26.3% (24.8–27.9)	
Obese	36.0% (34.7–37.3)	34.6% (32.9–36.3)	37.4% (35.9–38.9)	
**Mean WBC (1000 cells/ul)**				< 0.001 ^g^
Mean (95% confidence interval)	7.3 (7.2–7.4)	7.2 (7.1–7.3)	7.4 (7.3–7.5)	
**Year**				0.8 ^f^
2005–2006	20.4% (18.1–22.9)	20.2% (18.0–22.5)	20.6% (18.0–23.4)	
2007–2008	19.7% (17.4–22.3)	19.5% (17.4–21.8)	19.9% (17.3–22.8)	
2009–2010	19.2% (17.3–21.2)	19.4% (17.5–21.5)	18.9% (16.8–21.1)	
2011–2012	19.9% (17.8–22.3)	20.0% (17.7–22.6)	19.8% (17.6–22.3)	
2013–2014	20.8% (18.8–23.1)	20.8% (18.6–23.2)	20.8% (18.8–23.1)	

^a^ Participants were considered to have depressive symptoms if the PHQ-9 score was equal to or larger than 10

^b^ Participants were sexual minorities if they were gay/lesbian, bisexual, not sure about their sexual identities, or other sexual orientations

^c^ 40 was the median of participants’ age

^d^ Other races included Mexican American, Asian, and other races, including multi-racial

^e^ Poverty-income ratio (PIR) was used to categorize income: poverty (PIR < 1), low income (1.0 ≤ PIR < 2.0), middle income (2.0 ≤ PIR < 4.0), and high income (PIR ≥ 4.0)

^f^ Rao-Scott Chi-square test was used to calculate the *P*-value

^g^ t-test was used to calculate the *P*-value

In Table 2, we further stratified the sample by sexual minority status among males and females respectively. In the male population, the proportion of depressive symptoms differed significantly between heterosexual individuals (5.3%, 95% CI: 4.7–6.1) and sexual minorities (10.6%, 95% CI: 7.5–14.7). Similarly significant results were found in females: 9.4% (95% CI: 8.6–10.3) of heterosexual women had depressive symptoms, and the number was 23.7% (95% CI: 19.4–28.7) in sexual minority women.

**Table 2:** The weighted demographic characters of the study sample stratified by biological sex and sexual identity, NHANES 2005 to 2014

Variable	Male *n* = 6,880			Female *n* = 7,210		
	Heterosexual *n* = 6,541 95.2% (94.2–96.1)	Sexual minority ^b^ *n* = 339 4.8% (3.9–5.8)	*p*-value	Heterosexual *n* = 6,654 93.1% (92.3–93.8)	Sexual minority ^b^ *n* = 556 6.9% (6.2–7.7)	*p*-value
**Depressive symptoms **^**a**^			0.001 ^f^			0.001 ^f^
No	94.7% (93.9–95.3)	89.4% (85.3–92.5)		90.60% (89.7–91.4)	76.3% (71.3–80.6)	
Yes	5.3% (4.7–6.1)	10.6% (7.5–14.7)		9.4% (8.6–10.3)	23.7% (19.4–28.7)	
**Age **^**c**^			0.6 ^f^			0.001 ^f^
< 40	49.4% (47.6–51.2)	51.1% (43.8–58.4)		47.2% (45.4–49.0)	63.8% (58.8–68.5)	
> = 40	50.6% (48.8–52.4)	49.0% (41.6–56.2)		52.8% (51.0–54.6)	36.2% (31.5–41.2)	
**Race**			0.7 ^f^			0.01 ^f^
White	68.2% (65.1–71.1)	66.5% (60.3–72.2)		68.3% (64.9–71.4)	62.8% (57.2–68.0)	
Black	10.1% (8.8–11.6)	11.0% (8.1–14.8)		11.7% (10.2–13.7)	16.5% (12.8–21.1)	
Hispanic	5.2% (4.1–6.4)	6.3% (4.3–9.1)		5.4% (4.4–6.6)	4.6% (29.8–71.3)	
Other ^d^	16.5% (14.6–18.6)	16.2% (12.6–20.5)		14.6% (12.9–16.5)	16.1% (13.1–19.7)	
**Education**			0.05 ^f^			0.001 ^f^
B.A. and above	28.3% (26.1–30.6)	36.5% (28.9–44.8)		32.6% (30.1–35.2)	23.0% (18.4–28.3)	
A.A. and some college	31.3% (30.0–32.9)	30.7% (25.1–37.0)		35.0% (33.4–36.5)	35.2% (30.3–40.4)	
High school	24.4% (22.8–26.2)	17.6% (12.6–24.0)		19.6% (18.2–21.2)	22.3% (18.1–27.2)	
Did not complete high school	15.9% (14.4–17.5)	15.2% (11.4–20.1)		12.8% (11.4–14.3)	19.5% (16.1–23.5)	
**Income **^**e**^			0.4 ^f^			0.001 ^f^
High	39.0% (36.7–41.4)	35.1% (27.8–43.1)		38.0% (35.4–40.5)	23.9% (19.4–29.1)	
Middle	28.4% (26.7–30.0)	29.8% (24.9–35.3)		28.9% (27.2–30.6)	23.2% (19.0–28.0)	
Low	18.6% (17.3–20.0)	17.4% (12.6–23.5)		18.1% (16.9–19.4)	25.1% (20.8–30.0)	
Poverty	14.0% (12.6–15.6)	17.7% (13.7–22.5)		15.0% (13.5–16.7)	27.8% (24.2–31.7)	
**Smoking status**			0.1 ^f^			0.001 ^f^
Non-smokers	51.8% (49.8–53.7)	47.3% (40.0–54.8)		61.1% (59.1–63.1)	46.2% (41.3–51.2)	
Former smokers	21.8% (20.5–23.1)	19.2% (14.3–25.3)		17.8% (16.2–19.4)	16.6% (12.6–21.5)	
Current smokers	26.5% (24.8–28.2)	33.5% (26.7–41.0)		21.1% (19.5–22.9)	37.2% (33.1–41.5)	
**BMI**			0.01 ^f^			0.01 ^f^
Underweight	0.9% (0.7%-1.3)	1.3% (0.6–2.7)		2.1% (1.7–2.6)	3.5% (2.0–6.2)	
Normal	25.7% (24.1–27.3)	36.3% (30.7–42.2)		34.3% (32.6–36.1)	30.4% (24.9–36.4)	
Overweight	38.5% (37.1–40.0)	34.1% (28.7–40.0)		26.8% (25.2–28.4)	19.8% (15.9–24.4)	
Obese	34.9% (33.2–36.6)	28.3% (23.2–34.1)		36.7% (35.2–38.3)	46.3% (40.3–52.4)	
**Mean WBC (1000 cells/ul)**			0.01 ^g^			0.06 ^g^
Mean (95% confidence interval)	7.2 (7.1–7.3)	6.9 (6.6–7.1)		7.4 (7.3–7.5)	7.6 (7.4–7.9)	
**Year**			0.7 ^f^			0.2 ^f^
2005–2006	20.1% (17.9–22.6)	21.2% (15.0–29.1)		20.9% (18.3–23.7)	16.5% (12.3–21.7)	
2007–2008	19.7% (17.4–22.3)	16.1% (9.8–25.4)		20.0% (17.3–23.0)	18.3% (14.1–23.3)	
2009–2010	20.0% (17.5–21.8)	17.0% (11.8–23.1)		18.8% (16.8–21.1)	19.5% (15.0–25.0)	
2011–2012	19.8% (17.4–22.5)	23.5% (15.1–34.5)		19.8% (17.5–22.3)	20.6% (16.3–25.8)	
2013–2014	20.8% (18.5–23.2)	22.5% (17.0–29.2)		20.5% (18.4–22.8)	25.1% (20.6–30.2)	

^a^ Participants were considered to have depressive symptoms if the PHQ-9 score > 10

^b^ Participants were sexual minorities if they were gay/lesbian, bisexual, not sure about their sexual identities, or other sexual orientations

^c^ 40 was the median of participants’ age

^d^ Other races included Mexican American, Asian, and other races, including multi-racial

^e^ Poverty-income ratio (PIR) was used to categorize income: poverty (PIR < 1), low income (1.0 ≤ PIR < 2.0), middle income (2.0 ≤ PIR < 4.0), and high income (PIR ≥ 4.0)

^f^ Rao-Scott Chi-square test was used to calculate the *p*-value

^g^ *t*-test was used to calculate the *p*-value

To determine whether the inflammatory level was associated with depressive symptoms with regard to covariates, we examined the mean WBC count among subgroups stratified by sexual minorities, sex assigned at birth, and depressive symptoms. Results are summarized in Table 3. Among male heterosexual individuals, the mean WBC count was different between men who did not have depressive symptoms (7.20 per 1000 cells/ul, 95% CI: 7.11–7.28) and those who did (7.68 per 1000 cells/ul, 95% CI: 7.37–8.00). Although the confidence intervals of the point estimates overlapped, this did not preclude significance, especially when the point estimates were different from each other and the overlapping parts were relatively small [30]. Among male sexual minorities, similar differences were found, without statistical significance (Without depressive symptoms: 6.87 per 1000 cells/ul, 95% CI: 6.62–7.11 V.S. With depressive symptoms: 6.99 per 1000 cells/ul, 95% CI: 6.22 – 7.77; *p*-value = 0.74).

**Table 3:** The weighted demographic characters of the study sample stratified by biological sex, sexual identity, and depressive symptoms, NHANES 2005 to 2014

Variable	Male *n* = 6,880						Female *n* = 7,210					
	Heterosexual *n* = 6,541 95.2% (94.2–96.1)			Sexual minority ^b^ *n* = 339 4.8% (3.9–5.8)			Heterosexual *n* = 6,654 93.1% (92.3–93.8)			Sexual minority ^b^ *n* = 556 6.9% (6.2–7.7)		
	No depressive symptoms ^a^ n = 6,126	Depressive symptoms *n* = 415	*p*-value	No depressive symptoms ^a^ *n* = 305	Depressive symptoms *n* = 38	*p*-value	No depressive symptoms ^a^ *n* = 5,890	Depressive symptoms *n* = 764	*p*-value	No depressive symptoms ^a^ *n* = 433	Depressive symptoms *n* = 123	*p*-value
**Age **^**c**^			0.016			0.077			< 0.001			0.569
< 40	49.8% (47.9%-51.7%)	42.2% (36.7%-47.8%)		49.3% (41.5%-57.0%)	66.6% (47.2%-81.6%)		48.1% (46.2%-50.0%)	38.7% (34.7%-42.9%)		62.9% (56.7%-68.8%)	66.6% (55.6%-67.1%)	
> = 40	50.2% (48.3%-52.1%)	57.8% (52.2%-63.3%)		50.7% (43.0%-58.5%)	33.4% (18.4%-52.8%)		51.9% (50.0%-53.8%)	61.3% (57.1%-65.3%)		37.1% (31.2%-43.3%)	33.4% (23.9%-44.4%)	
**Race**			0.101			0.468			< 0.001			0.089
White	68.3% (65.3%-71.3%)	65.6% (59.1%-71.6%)		67.5% (60.9%-73.5%)	58.0% (39.4%-74.6%)		67.8% (65.5%-71.9%)	63.2% (75.6%-68.5%)		61.5% (55.2%-67.4%)	66.9% (57.7%-75.0%)	
Black	10.0% (8.7%-11.4%)	12.9% (9.4%-17.6%)		11.0% (0.8%-14.9%)	11.4% (5.1%-23.7%)		11.4% (9.6%-13.3%)	15.4% (12.6%-18.5%)		51.8% (11.9%-20.7%)	18.8% (13.1%-36.2%)	
Hispanic	5.1% (4.1%-6.4%)	6.6% (4.6%-9.3%)		5.7% (3.9%-8.2%)	11.6% (4.2%-28.4%)		5.1% (4.2%-6.2%)	7.9% (5.6%-11.0%)		4.6% (2.8%-7.3%)	4.9% (2.3%-9.8%)	
Other ^d^	16.6% (14.7%-18.7%)	14.9% (11.3%-19.4%)		15.8% (12.2%-20.3%)	18.9% (7.7%-39.5%)		14.7% (13.0%-16.6%)	13.6% (10.8%-17.0%)		18.2% (14.6%-22.4%)	9.4% (5.3%-16.2%)	
**Education**			< 0.001			0.002			< 0.001			0.246
B.A. and above	29.3% (27.1%-31.5%)	11.3% (7.6%-16.4%)		39.8% (31.8%-48.5%)	8.0% (1.8%-28.8%)		34.6% (32.0%-37.3%)	13.9% (10.4%-18.3%)		25.3% (20.0%-31.4%)	15.5% (8.6%-26.4%)	
A.A. and some college	31.2% (29.6%-32.8%)	34.6% (39.4%-40.3%)		30.8% (25.0%-21.2%)	30.4% (15.9%-50.2%)		34.7% (33.0%-36.4%)	37.7% (33.1%-42.6%)		34.7% (29.8%-40.0%)	36.6% (25.9%-48.7%)	
High school	24.1% (22.4%-25.9%)	30.6% (25.9%-35.6%)		15.7% (11.0%-21.8%)	33.6% (18.5%-53.0%)		19.1% (17.6%-20.7%)	24.8% (20.9%-29.2%)		21.5% (17.4%-26.3%)	24.9% (16.7%-35.5%)	
Did not complete high school	15.5% (14.1%-17.1%)	23.6% (19.2%-28.5%)		13.7% (9.8%-18.9%)	28.0% (14.9%-46.4%)		11.6% (10.3%-13.1%)	23.5% (20.0%-27.6%)		18.5% (14.8%-22.9%)	22.9% (15.6%-32.3%)	
**Income **^**e**^			< 0.001			< 0.001			< 0.001			0.013
High	40.0% (37.7%-42.5%)	20.4% (15.0%-27.2%)		28.9% (31.2%-47.2%)	2.9% (0.6%-11.9%)		39.9% (37.4%-42.5%)	18.7% (15.2%-22.9%)		26.2% (20.8%-32.3%)	16.7% (10.0%-26.5%)	
Middle	28.8% (27.0%-30.5%)	21.2% (16.2%-27.3%)		30.0% (24.4%-36.1%)	28.9% (13.7%-51.0%)		29.4% (27.7%-31.1%)	34.8% (2.00%-28.0%)		25.8% (20.7%-31.7%)	14.7% (8.4%-24.5%)	
Low	18.0% (16.7%-19.4%)	29.8% (25.2%-35.0%)		16.9% (12.6%-22.3%)	21.8% (7.8%-47.8%)		17.1% (15.9%-18.4%)	27.9% (24.2%-31.9%)		34.7% (19.0%-29.3%)	29.6% (19.9%-41.6%)	
Poverty	13.2% (11.9%-14.7%)	28.6% (24.5%-33.0%)		14.3% (10.6%-18.9%)	46.5% (26.3%-67.9%)		13.5% (12.1%-15.0%)	29.6% (25.2%-34.4%)		24.3% (20.2%-28.9%)	39.0% (30.9%-47.8%)	
**Smoking status**			< 0.001			0.002			< 0.001			0.004
Non-smokers	52.8% (50.9%-54.7%)	33.1% (26.1%-40.9%)		49.9% (42.0%-57.7%)	25.8% (13.0%-44.6%)		63.2% (61.1%-65.2%)	41.2% (36.8%-45.7%)		51.2% (45.2%-57.1%)	30.3% (20.7%-41.9%)	
Former smokers	21.7% (20.4%-23.1%)	22.7% (18.0%-28.3%)		20.3% (15.1%-26.7%)	10.0% (2.6%-31.5%)		18.0% (16.5%-19.7%)	15.3% (11.8%-19.8%)		16.7% (12.1%-22.5%)	16.2% (9.0%-27.3%)	
Current smokers	25.5% (23.8%-27.2%)	44.2% (38.0%-50.6%)		29.9% (23.0%-37.8%)	64.2% (44.8%-79.9%)		18.8% (17.2%-20.5%)	43.5% (38.4%-48.7%)		32.2% (27.6%-37.1%)	53.6% (43.7%-63.2%)	
**BMI**			0.148			0.597			< 0.001			0.420
Underweight	1.0% (0.7%-1.3%)	0.8% (0.3%-2.0%)		1.3% (0.6%-2.9%)	1.0% (0.1%-7.3%)		2.2% (1.7%-2.7%)	1.8% (0.9%-3.8%)		3.5% (1.9%-6.5%)	3.5% (0.8%-13.3%)	
Normal	25.5% (23.9%-27.1%)	29.4% (23.2%-36.4%)		35.7% (30.2%-41.6%)	41.3% (21.4%-64.5%)		35.6% (338%-37.5%)	21.8% (18.8%-25.2%)		30.1% (23.8%-37.2%)	31.2% (22.7%-41.3%)	
Overweight	38.9% (37.3%-40.4%)	31.9% (26.4%-37.8%)		35.3% (30.0%-41.1%)	23.8% (9.8%-47.1%)		26.9% (25.3%-28.5%)	25.7% (21.6%-30.4%)		21.8% (17.4%-27.0%)	13.4% (7.8%-22.1%)	
Obese	34.7% (32.9%-36.6%)	38.0% (32.0%-44.5%)		27.6% (27.3%-33.8%)	34.0% (17.8%-55.2%)		35.3% (33.7%-37.0%)	50.6% (45.8%-55.4%)		44.6% (34.3%-25.1%)	51.8% (41.4%-62.2%)	
**Mean WBC (1000 cells/ul)**			0.002			0.74			< 0.001			0.005
Mean (95% confidence interval)	7.20 (7.11–7.28)	7.68 (7.37 – 8.00)		6.87 (6.62–7.11)	6.99 (6.22 – 7.77)		7.35 (7.26–7.44)	7.96 (7.76 – 8.15)		7.47 (7.21 – 7.73)	8.20 (7.71 – 8.69)	
**Year**			0.321			0.351			0.009			0.850
2005–2006	20.3% (18.0%-22.7%)	17.0% (13.0%-22.1%)		21.3% (15.1%-29.3%)	20.1% (8.0%-24.1%)		21.5% (18.8%-24.5%)	14.4% (10.7%-19.0%)		17.0% (12.4%-23.0%)	14.7% (8.4%-22.5%)	
2007–2008	19.7% (17.4%-22.3%)	19.5% (13.8%-26.7%)		17.4% (10.6%-27.1%)	5.5% (1.9%-15.2%)		19.7% (17.1%-22.6%)	22.7% (18.0%-28.2%)		19.3% (14.6%-25.0%)	15.2% (9.3%-23.6%)	
2009–2010	19.4% (17.3%-21.7%)	22.2% (17.5%-27.8%)		15.8% (11.2%-21.9%)	24.2% (12.8%-41.0%)		18.8% (16.6%-21.2%)	19.4% (16.5%-22.6%)		18.9% (14.0%-25.0%)	21.3% (13.7%-31.5%)	
2011–2012	19.6% (17.2%-22.3%)	34.8% (17.8%-30.9%)		23.1% (14.4%-34.7%)	26.9% (13.4%-46.7%)		19.8% (14.4%-22.5%)	19.0% (15.0%-23.8%)		19.9% (16.0%-24.4%)	32.1% (13.0%-37.7%)	
2013–2014	20.9% (18.7%-23.4%)	17.5% (12.5%-23.9%)		22.4% (17.3%-28.6%)	23.2% (9.0%-48.1%)		20.1% (17.9%-22.6%)	14.5% (20.7%-28.8%)		24.9% (20.3%-30.1%)	25.7% (16.3%-38.2%)	

^a^ Participants were considered to have depressive symptoms if the PHQ-9 score was > 10

^b^ Participants were sexual minorities if they were gay/lesbian, bisexual, not sure about their sexual identities, or other sexual orientations

^c^ 40 was the median of participants’ age

^d^ Other races included Mexican American, Asian, and other races, including multi-racial

^e^ Poverty-income ratio (PIR) was used to categorize income: poverty (PIR < 1), low income (1.0 ≤ PIR < 2.0), middle income (2.0 ≤ PIR < 4.0), and high income (PIR ≥ 4.0)

^f^ Rao-Scott Chi-square test was used to calculate the *P*-value

^g^ t-test was used to calculate the *P*-value

Among females, differences in mean WBC count between women who had depressive symptoms and those who did not were both significantly different among heterosexuals (Without depressive symptoms: 7.35 per 1000 cells/ul, 95% CI: 7.26–7.44 V.S. With depressive symptoms: 7.96 per 1000 cells/ul, 95% CI: 7.76 – 8.15; *p*-value < 0.0001) and sexual minorities respectively (Without depressive symptoms: 7.47 per 1000 cells/ul, 95% CI: 7.21–7.73 V.S. With depressive symptoms: 8.20 per 1000 cells/ul, 95% CI: 7.71 – 8.69; *p*-value < 0.0001). These results are shown in Table 3.

**Fig. 1:**
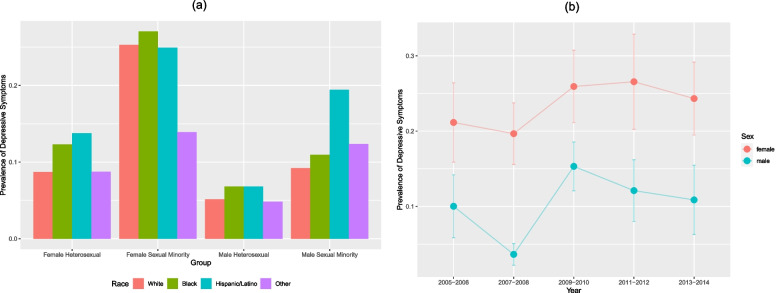
**a** Prevalence of depressive symptoms among heterosexual and sexual minority populations, stratified by biological sex: NHANES 2005 to 2014; **b** Prevalence of depressive symptoms among females and males, stratified by survey year: NHANES 2005 to 2014

We also performed a descriptive analysis considering the race of the sample. Black sexual minority females had the highest rate of depressive symptoms (19.4%, 95% CI: 7.72–40.98) compared with male heterosexual individuals in other racial groups (4.8%, 95% CI: 3.73–6.17) (Fig. 1a). Regarding survey years, females had a higher proportion of depressive symptoms in each of the survey years, and the confidence intervals between males and females did not overlap (Fig. 1b).

### Multiple logistic regression models

We examined the association between sexual minority status and depressive symptoms using two multiple logistic regression models. In the first model we treated sexual minority status as a binary variable, and found that people who identified as sexual minorities had 1.79 (95% CI: 1.42 – 2.17) times the odds of having depressive symptoms compared to people who were not sexual minorities. Both models were assessed with F-adjusted mean residual test to confirm the goodness of fit (Model 1: F-adjusted test statistic = 0.936, Prob > F = 0.499; Model 2: F-adjusted test statistic = 0.800, Prob > F = 0.617).

In the second model, we further stratified sexual minority status into 4 categories, gay/lesbian, bisexual, not sure about sexual identity, and other sexual identities. Among different sexual minority groups, we found that people who were unsure about their sexual identities had the highest odds of having depressive symptoms compared to heterosexual individuals, but the confidence intervals overlapped with those of other sexual minority groups (2.56, 95% CI: 1.40–4.68). The results were included in Table 4.

**Table 4:** The association between sexual minority status/sexual orientation groups and depressive symptoms, NHANES 2005 to 2014

	**Main Exposer of Interests**	**Odds Ratio (OR)**	**Standard Error (SE)**	**95% Confidence Interval (CI)**
**Model 1**^**a**^	**Sexual Minority Status **^**c**^			
	No (Reference)	-	-	-
	Yes	2.23***	0.28	(1.74, 2.85)
**Model 2 **^**b**^	**Sexual Orientations**			
	Heterosexual (Reference)	-	-	-
	Gay/lesbian	1.71*	0.41	(1.07, 2.75)
	Bisexual	2.48***	0.41	(1.78, 3.46)
	Not sure	2.60**	0.81	(1.40, 4.83)
	Others	1.71	0.76	(0.71, 4.12)

^*^ *p*-value < 0.05

^**^ *p*-value < 0.01

^***^ *p*-value < 0.001

^a^ Model 1 was adjusted for sex, age, race, education level, income, smoking status, BMI, WBC, and survey year

^b^ Model 2 was adjusted for sex, age, race, education level, income, smoking status, BMI, WBC, and survey year

^c^ Participants were sexual minorities if they were gay/lesbian, bisexual, not sure about their sexual identities, or other sexual orientations

## Discussion

Our study found that female and sexual minority status were both independently associated with having depressive symptoms. Specifically, females had a higher proportion of depressive symptoms compared to males during all survey years. Within the sexual minority population, subjects who were not sure about their sexual identities had the highest odds of having depressive symptoms. In the subgroup analysis considering race, sex, and sexual minority status, Black female sexual minority members had the highest rate of depressive symptoms.

Among male heterosexual individuals, the mean WBC count was significantly different between participants with and without depressive symptoms. Among male sexual minorities, a similar but nonsignificant difference was found. Among females, the mean WBC count was significantly different between people with and without depressive symptoms, and the results also applied to both heterosexual and sexual minority subjects.

Our study’s results agree with the findings that women are at higher risk of having depressive symptoms compared with men [31]. Certain reproductive-related hormonal changes might be the reason for this increased risk [32]. It is also possible that the difference is caused by sex-based reporting differences, where females are more likely to report depression related symptoms compared with males [33, 34]. Other female specific social determinants of depression related symptoms included intimate partner violence, unplanned pregnancy, male gender preference and poor relationship with in-laws [35].

The observed increased risk of depressive symptoms in the sexual minority groups was in line with previous studies [4]. Negative experiences and stressors such as discrimination, victimization, harassment, abuse, increased stress, and lower social and family support may contribute to differing depression rates in sexual minorities compared to heterosexual counterparts [4, 36].

Few studies thus far have included people who were not sure about their sexual orientation in the sexual minority group in a nationally representative sample in the US; thus, the health risk of sexual minority groups might be underestimated. Our study found that people with uncertainty in sexual orientation had the highest odds of suffering from depressive symptoms compared with gay/lesbian, bisexual, and other sexual orientation groups. Uncertainty in general is associated with strong emotions [37] and psychological maladjustment [38]. Several studies indicated the association between uncertainty in sexual orientations and adverse mental health outcomes in the adolescent and early adulthood population [39, 40]. Our study extended this association into the adult population. This is reasonable because the exploration of relationships, although essential and meaningful, increased stressors in both the adult and adolescent population and led to depressive and anxious symptoms [41]. Researchers have also found that identity uncertainty is associated with increased levels of internalized homophobia in sexual minorities [41, 42]. This is possibly because sexual minorities may be more likely to internalize society’s negative attitudes toward homosexuality to define themselves [42].

In the subgroup analysis, we found that Black female sexual minorities had the highest rate of depressive symptoms. This could be partially explained by the disadvantages of poverty and more limited education in this subgroup [43]. According to the minority stress model, sexual minority individuals were embedded with more stigma, prejudice, and discrimination compared with heterosexual individuals [44]. It is likely that systemic racism faced by the Black population further worsened the mental health conditions of this subpopulation [45, 46]. From an intersectionality perspective, the subgroup of Black female sexual minorities are faced with overlapping and interdependent systems of discrimination or disadvantage, leading to their health inequality conditions [47, 48].

Our study found that the mean WBC count was significantly different between people who had and those who did not have depressive symptoms among all combinations of gender and sexual orientation except male sexual minorities. Although the confidence intervals for the point estimate of the mean WBC overlapped among subgroups, it did not preclude statistical significance [30, 49]. Together with confidence intervals, point estimates also provided valuable information on health science research [50–53]. In a systematic review examining the effect of acute experimental inflammation on negative biases, researchers found evidence for experimental inflammation on negative biases in emotional processing, and negative biases in emotional processing are thought to be of central importance in the development and maintenance of depression [54]. Another review also suggested that chronic stress is associated with increased inflammatory activity and enhanced attentional processing of negative information [55]. These findings indicate that systematic inflammation and affective-cognitive processes may be intertwined and might potentiate one another’s impact on depressive symptoms. However, we did not find a significant difference in mean WBC count between depressed and non-depressed people among male sexual minorities. A possible explanation was that people with and without depressive symptoms within this subpopulation experienced similar stress levels, compared with other subgroups. Although the cause of this is not known, it might be related to the effects of social stigma surrounding homosexuality in males or the living styles in which sexual minority males differ from other subgroups [56]. The mechanisms also might include discrimination, insufficient social support, and HIV related stigma [57, 58].

Furthermore, unequal health care systems faced by sexual and gender minority individuals (SGM) might drive the elevated WBC count in the target population. In the Survey of the Health of Wisconsin (SHOW) from 2014 to 2016, researchers showed that LGB individuals are more likely to delay health care than non-LGB individuals, and trans respondents were more likely to report poor quality of care than non-LGBT. These delays in and low-quality of health care can lead to an increased level of inflammation biomarkers [59–61].

This study has several limitations. First, several factors, including lifestyles, diseases, gender minority and transgender information, might also affect depression levels and the mean WBC count. However, they were not reported in the questionnaire of the national sample, and therefore unavailable in this work. Second, due to the nature of the cross-sectional study, the results were vulnerable to recall bias, and causality may not be inferred, either. Future research should construct a prospective cohort to control for possible confounders and include early childhood experience as a potential influence on the outcomes. Third, although the study was descriptive in nature, the stratification analysis was still vulnerable to sample size and bias. The subgroup sample size is relatively small and after weighting the sample, the confidence intervals were relatively large even though the study was designed to be nationally representative. Fourth, although social events were associated with having depressive symptoms since 2014, more recent data were not available on all the variables in the dataset. We encourage further research incorporating future study waves to capture the more recent trends. Future studies can use regression methods to control for confounding and testing the effect measure modification on both the multiplicative and additive scale.

## Conclusion

Combining sex, race, and sexual minority status, we found that Black, sexual minority females had the highest rate of depressive symptoms. Within subgroups of the sexual minority population, people who were not sure about their sexual identities had the highest odds of having depressive symptoms. Furthermore, the mean WBC count was significantly higher in people with depressive symptoms in all combinations of gender and sexual orientation except in male sexual minorities. Future research should investigate the social and biological mechanisms that may contribute to such differences. Moreover, we encourage clinicians and public health workers to pay close attention to high-risk populations with depressive symptoms.

## Supplementary Information

**Additional file 1.** Directed acyclic graph for the study. 

**Additional file 2:** **Supplementary Table. **The association betweensexual minority status/sexual orientation groups and PHQ-9 scores, NHANES 2005to 2014.

## Data Availability

The datasets generated during and/or analyzed during the current study are available in the NHANES repository, https://wwwn.cdc.gov/nchs/nhanes/Default.aspx [25].
