# Supplementary figures and images for: The descriptive analysis of depressive symptoms and White Blood Cell (WBC) count between the sexual minorities and heterosexual identifying individuals in a nationally representative sample: 2005–2014
Source: BMC Public Health. 2023 Feb 9;23:294. doi: 10.1186/s12889-022-14847-6 (PMC9909981; doi:10.1186/s12889-022-14847-6)

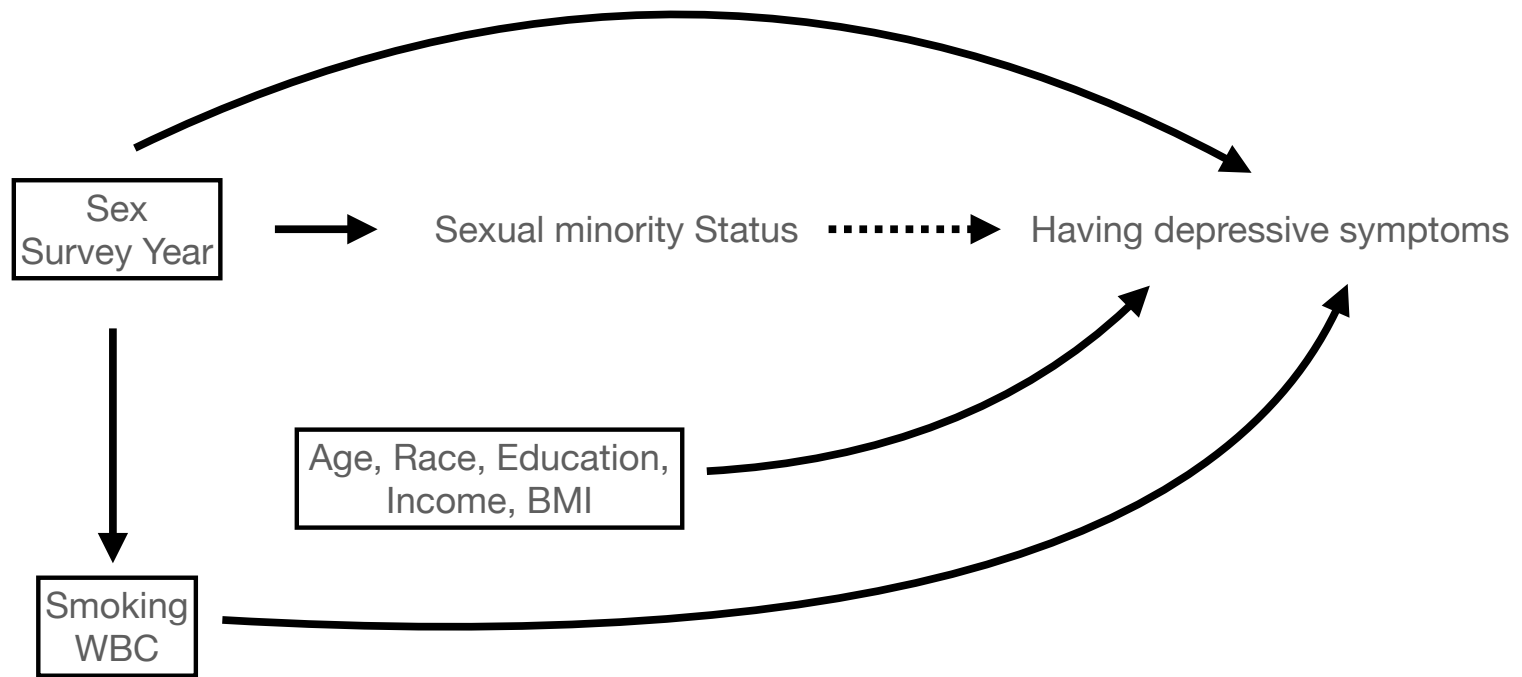

**Directed acyclic graph for the study**

Supplement: Supplementary file 1 — Additional file 1. Directed acyclic graph for the study. [file 12889_2022_14847_MOESM1_ESM.pdf]
