# Supplementary material for: The descriptive analysis of depressive symptoms and White Blood Cell (WBC) count between the sexual minorities and heterosexual identifying individuals in a nationally representative sample: 2005–2014
Source: BMC Public Health. 2023 Feb 9;23:294. doi: 10.1186/s12889-022-14847-6 (PMC9909981; doi:10.1186/s12889-022-14847-6)
Supplement: Supplementary file 2 — Additional file 2: Supplementary Table. The association betweensexual minority status/sexual orientation groups and PHQ-9 scores, NHANES 2005to 2014. [file 12889_2022_14847_MOESM2_ESM.docx]

**Supplementary Table. The association between sexual minority status/sexual orientation groups and PHQ-9 scores, NHANES 2005 to 2014**

|  | **Main Exposer of Interests** | **Coefficient** | **Standard Error (SE)** | **95% Confidence Interval (CI)** |
| --- | --- | --- | --- | --- |
| **Model 1^a^** | **Sexual Minority Status ^c^** |  |  |  |
|  | No (Reference) | - | - | - |
|  | Yes | 1.45*** | 0.22 | (1.01, 1.90) |
| **Model 2^b^** | **Sexual Orientations** |  |  |  |
|  | Heterosexual (Reference) | - | - | - |
|  | Gay/lesbian | 0.98 ** | 0.34 | (0.30, 1.66) |
|  | Bisexual | 1.95 *** | 0.30 | (1.36, 2.55) |
|  | Not sure | 1.16 * | 0.56 | (0.05, 2.28) |
|  | Others | 0.88 | 0.75 | (-0.61, 2.38) |

* *p*-value < 0.05, ** *p*-value < 0.01, *** *p*-value < 0.001

1. Model 1 was adjusted for sex, age, race, education level, income, smoking status, BMI, WBC, and survey year
2. Model 2 was adjusted for sex, age, race, education level, income, smoking status, BMI, WBC, and survey year
3. Participants were sexual minorities if they were gay/lesbian, bisexual, not sure about their sexual identities, or other sexual orientations.
